# Dataset on the structure and thermodynamic and dynamic stability of Mo_2_ScAlC_2_ from experiments and first-principles calculations

**DOI:** 10.1016/j.dib.2016.12.046

**Published:** 2016-12-29

**Authors:** Martin Dahlqvist, Rahele Meshkian, Johanna Rosen

**Affiliations:** Department of Physics, Chemistry and Biology (IFM), Linköping University, Linköping SE-581 83, Sweden

## Abstract

The data presented in this paper are related to the research article entitled “Theoretical stability and materials synthesis of a chemically ordered MAX phase, Mo_2_ScAlC_2_, and its two-dimensional derivate Mo_2_ScC” (Meshkian et al. 2017) [1]. This paper describes theoretical phase stability calculations of the MAX phase alloy Mo_x_Sc_3-x_AlC_2_ (*x*=0, 1, 2, 3), including chemical disorder and out-of-plane order of Mo and Sc along with related phonon dispersion and Bader charges, and Rietveld refinement of Mo_2_ScAlC_2_. The data is made publicly available to enable critical or extended analyzes.

**Specifications Table**

**Table:** 

Subject area	Physics, Materials science
More specific subject area	Phase stability predictions,
Type of data	Tables, Figures, Text file
How data was acquired	Density functional theory calculations using VASP 5.3.3, phonon dispersion using Phonopy 1.9.1, and atom charges using Bader charge analysis version 0.95a.
	*θ-2θ* X-ray diffraction (XRD) measurements were performed on the samples using a diffractometer (Rikagu Smartlab, Tokyo, Japan), with Cu-K_*α*_ radiation (40 kV and 44 mA). The scans were recorded between 3° and 120° with step size of 0.02° and a dwell time of 7 s.
Data format	Raw, Analyzed
Experimental factors	N/A
Experimental features	For synthesis of Mo_2_ScAlC_2_, elemental powders of Mo, Sc, Al and graphite were mixed in an agate mortar, put in an alumina crucible, and placed into a sintering furnace where it was heated up to 1700 °C and kept at that temperature for 30 min. Structural characterization was performed using X-ray diffraction (XRD), and for complementary structural and compositional analysis high-resolution scanning transmission electron microscopy (HRSTEM) measurement were carried out. See Ref. [1] for further information.
Data source location	Linköping, Sweden
Data accessibility	Data are available with this article.

**Value of the data**

• This data allows other researchers to calculate and predict the phase stability of new compounds within the quaternary Mo-Sc-Al-C system and related subsystem.
• The data presents refined/calculated structures that can be used as input for further theoretical evaluation of properties.
• The structural information can also be used for interpretation and phase identification of, e.g., attained experimental XRD, (S)TEM, and electron diffraction data.

## 1 Data

**Fig. 1:**
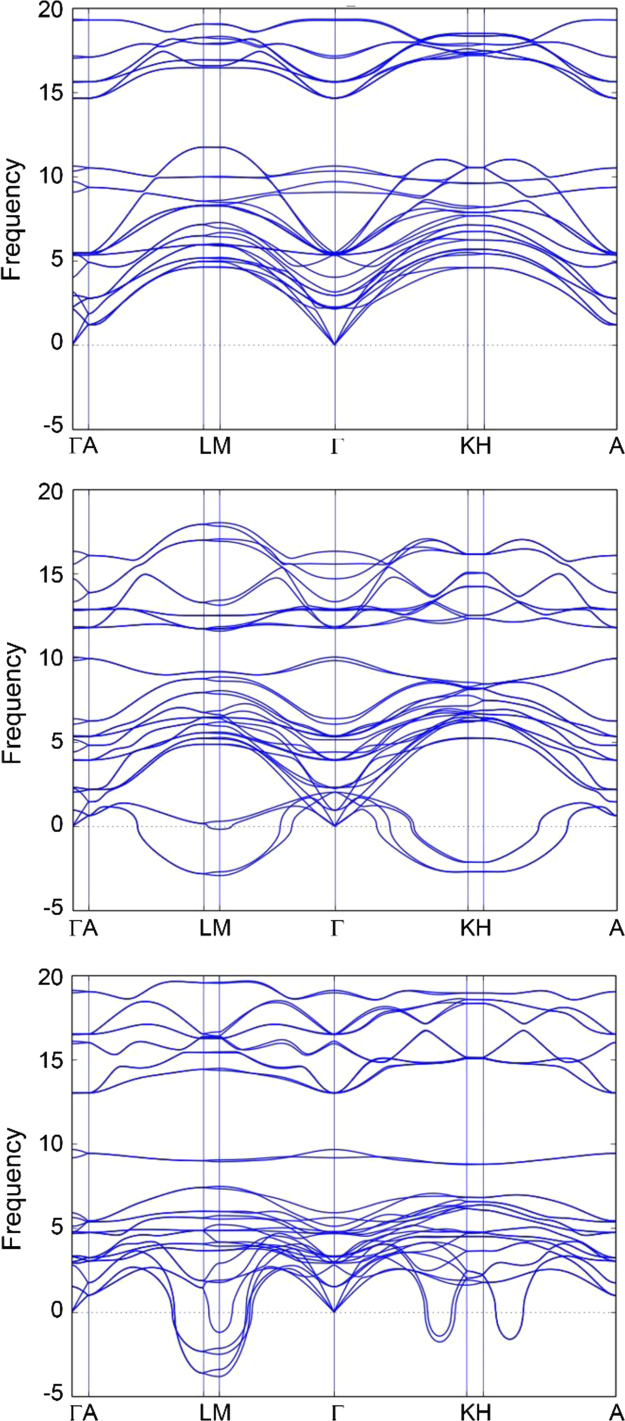
Calculated phonon dispersion for (a) Mo_2_ScAlC_2_, (b) Sc_3_AlC_2_, and (c) Mo_3_AlC_2_.

**Fig. 2:**
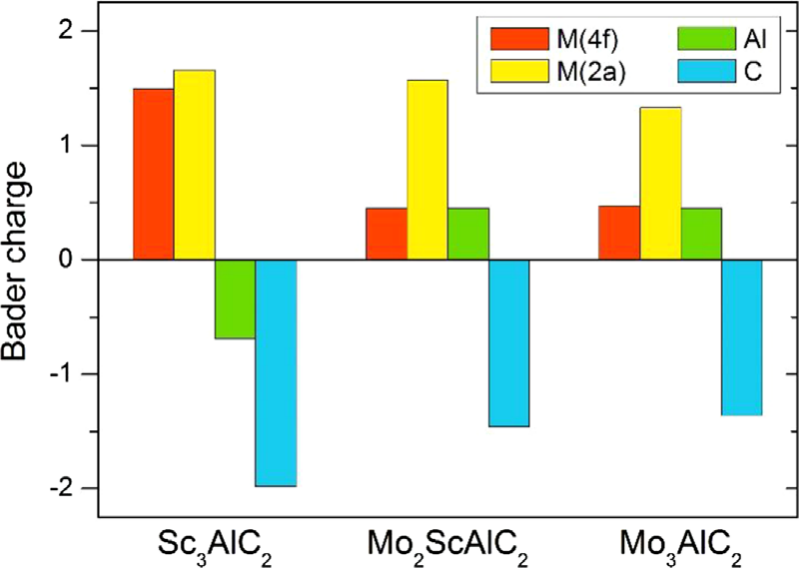
Calculated charge for atoms in Sc_3_AlC_2_, Mo_2_ScAlC_2_, and Mo_3_AlC_2_ using Bader analysis.

The dataset of this paper provides information for calculated phases within the quaternary Mo-Sc-Al-C system and data obtained from refinement of the XRD pattern. Table 1 provides calculated lattice parameters, formation enthalpy, and equilibrium simplex for the chemically ordered nanolaminates Mo_2_ScAlC_2_ and Sc_2_MoAlC_2_ with different atomic stacking sequences (described in detail in Fig. 7(a) in Ref. [2]). Table 2 provides information for all considered competing phases within the quaternary system. Fig. 1 show calculated phonon spectra for Mo_2_ScAlC_2_ of order A and its corresponding end members Sc_3_AlC_2_ and Mo_3_AlC_2_. Fig. 2 depicts calculated Bader charges of atoms in Mo_x_Sc_3-x_AlC_2_ (*x*=0, 2, 3). Table 3 shows the data obtained from refinement of the XRD pattern, see Ref. [1]; Lattice vectors *a*, *b* and *c* for the majority phase Mo_2_ScAlC_2_ are 3.033, 3.033 and 18.775 Å, respectively.

## 2 Experimental design, materials and methods

First-principles calculations were performed by means of density functional theory (DFT) and the projector augmented wave method [3], [4] as implemented within the Vienna ab-initio simulation package (VASP) 5.3.3 [5], [6], [7]. We adopted the non-spin polarized generalized gradient approximation (GGA) as parameterized by Perdew–Burke–Ernzerhof (PBE) [8] for treating electron exchange and correlation effects. A plane-wave energy cut-off of 400 eV was used and for sampling of the Brillouin zone we used the Monkhorst–Pack scheme [9]. The calculated total energy of all phases is converged to within 0.5 meV/atom with respect to k-point sampling and structurally optimized in terms of unit-cell volumes, c/a ratios (when necessary), and internal parameters to minimize the total energy.

Chemically disordered of Sc and Mo in Mo_x_Sc_3-x_AlC_2_ have been modelled using the special quasi-random structure (SQS) method [10], [11] on supercells of 4×4×1 *M*_3_*AX*_2_ unit cells, with a total of 96 *M*-sites, respectively. Convergence tests with respect to total energy show that these sizes are appropriate to use, based on an energy of the 4×4×1 unit cells being within 2 meV/atom compared to larger supercells.

Evaluation of phase stability was performed by identifying the set of most competing phases at a given composition, i.e. equilibrium simplex, using a linear optimization procedure [11], [12] including all competing phases in the system. A phase is considered thermodynamically stable when its energy is lower than the set of most competing phases, and when there is no imaginary frequencies in phonon spectra, i.e. an indicated dynamic stability. The approach has been proven successful to confirm already experimentally known MAX phases as well as to predict the existence of new ones [2], [13], [14].

Dynamical stability of the chemically ordered Mo_*x*_Sc_3-*x*_AlC_2_ (*x*=0, 2, 3) structures was evaluated by phonon calculations of 4×4×1 supercells using density functional perturbation theory and as implemented in the PHONOPY code, version 1.9.1 [15], [16]. Calculated charges were obtained using Bader charge analysis, version 0.95a [17].

The synthesis of Mo2ScAlC2 were carried out by mixing elemental powders of Mo, Sc, Al and graphite in an agate mortar, put in an alumina crucible, and placed into a sintering furnace where it was heated up to 1700 °C and kept at that temperature for 30 min.

*θ-2θ* X-ray diffraction (XRD) measurements were performed on the samples using a diffractometer (Rikagu Smartlab, Tokyo, Japan), with Cu-K_*α*_ radiation (40 kV and 44 mA). The scans were recorded between 3° and 120° with step size of 0.02^°^ and a dwell time of 7 s. XRD pattern was analyzed by Rietveld refinement using FULLPROF code [18], where 5 backgrounds parameters, scale factors, *X* and *Y* profile parameters, lattice parameters, atomic positions, the overall B-factor and the occupancies for the main as well as the impurity phases were fitted.

## Funding sources

J. R. acknowledges funding from the Swedish Research Council (VR) under Grant no. 621-2012-4425 and 642-2013-8020, from the Knut and Alice Wallenberg (KAW) Foundation, and from the Swedish Foundation for Strategic Research (SSF) through the synergy grant FUNCASE. All calculations were carried out using supercomputer resources provided by the Swedish National Infrastructure for Computing (SNIC) at the National Supercomputer Centre (NSC), the High Performance Computing Center North (HPC2N), and the PDC Center for High Performance Computing.

## Figures and Tables

**Table 1:** Calculated lattice parameters, equilibrium total energy *E*_0_ in eV per formula unit, formation enthalpy Δ*H*_cp_ in meV per atom, and identified equilibrium simplex for Mo_2_ScAlC_2_ and Sc_2_MoAlC_2_. For comparison the corresponding end members Mo_3_AlC_2_ and Sc_3_AlC_2_ are also included.

Phase	Order	*a* (Å)	*c* (Å)	*E*_0_ (eV/fu)	Δ*H*_cp_ (meV/atom)	Equilibrium simplex
Mo_3_AlC_2_		3.0716	18.541	−54.830	+141	C, Mo_3_Al
Mo_2_ScAlC_2_	A	3.0619	19.072	−52.431	—24	(Mo_2/3_Sc_1/3_)_2_AlC, MoC, ScC_0.875_, Mo
Mo_2_ScAlC_2_	B	3.0774	19.252	−51.972	+53	(Mo_2/3_Sc_1/3_)_2_AlC, MoC, ScC_0.875_, Mo
Mo_2_ScAlC_2_	C	3.1622	18.789	−51.601	+114	(Mo_2/3_Sc_1/3_)_2_AlC, MoC, ScC_0.875_, Mo
Mo_2_ScAlC_2_	D	3.1771	18.865	−51.505	+130	(Mo_2/3_Sc_1/3_)_2_AlC, MoC, ScC_0.875_, Mo
Mo_2_ScAlC_2_	E	3.1271	19.054	−51.348	+157	(Mo_2/3_Sc_1/3_)_2_AlC, MoC, ScC_0.875_, Mo
Mo_2_ScAlC_2_	F	3.1221	19.109	−51.663	+104	(Mo_2/3_Sc_1/3_)_2_AlC, MoC, ScC_0.875_, Mo
Mo_2_ScAlC_2_	disorder	3.1252	18.861	−51.767	+87	(Mo_2/3_Sc_1/3_)_2_AlC, MoC, ScC_0.875_, Mo
Sc_2_MoAlC_2_	A	3.1798	19.819	−48.262	+28	(Mo_2/3_Sc_1/3_)_2_AlC, Sc_3_AlC, Sc_3_C_4_
Sc_2_MoAlC_2_	B	3.1808	19.845	−48.071	+60	(Mo_2/3_Sc_1/3_)_2_AlC, Sc_3_AlC, Sc_3_C_4_
Sc_2_MoAlC_2_	C	3.1886	19.696	−47.842	+98	(Mo_2/3_Sc_1/3_)_2_AlC, Sc_3_AlC, Sc_3_C_4_
Sc_2_MoAlC_2_	D	3.1892	19.770	−47.864	+94	(Mo_2/3_Sc_1/3_)_2_AlC, Sc_3_AlC, Sc_3_C_4_
Sc_2_MoAlC_2_	E	3.2279	19.802	−47.453	+162	(Mo_2/3_Sc_1/3_)_2_AlC, Sc_3_AlC, Sc_3_C_4_
Sc_2_MoAlC_2_	F	3.1898	19.700	−47.779	+108	(Mo_2/3_Sc_1/3_)_2_AlC, Sc_3_AlC, Sc_3_C_4_
Sc_2_MoAlC_2_	disorder	3.2251	19.335	−48.088	+57	(Mo_2/3_Sc_1/3_)_2_AlC, Sc_3_AlC, Sc_3_C_4_
Sc_3_AlC_2_		3.3170	20.885	−43.406	+155	Sc_3_AlC, Sc_3_C_4_, ScAl_3_C_3_

**Table 2:** Structural information and calculated total energy for competing phases considered within the quaternary Mo-Sc-Al-C system.

Phase	Prototype structure	Pearson symbol	Space group	*V* (Å^3^/uc)	*a*	*b*	*c*	*E*_0_ (eV/fu)
					(Å)	(Å)	(Å)	
Mo	W	*cI*2	*Im*-3*m* (229)	15.92	3.169			−10.850
Mo	Cu	*cF*4	*Fm*-3*m* (225)	16.15	4.012			−10.431
Mo	Mg	*hP*2	*P*6_3_/*mmc* (194)	32.57	2.774		4.887	−10.414
Sc	Mg	*hP*2	*P*6_3_/*mmc* (194)	49.25	3.321		5.157	−6.333
Sc	Sc	*hP*6	*P*6_1_22 (178)	148.75	3.242		16.342	−6.201
Sc	Np	*tP*4	P4/nmm (129)	100.35	5.367		3.484	−6.223
Al	Cu	*cF*4	*Fm*-3*m* (225)	66.00	4.041			−3.745
Al	Mg	*hP*2	*P*6_3_/*mmc* (194)	33.28	2.856		4.712	−3.712
Al	W	*cI*2	*Im*-3*m* (229)	16.93	3.235			−3.649
C	C (graphite)	*hP*4	*P*6_3_/*mmc* (194)	38.14	2.464		7.250	−9.225
Al_4_C_3_	Al_4_C_3_	*hR*21	*R*-3*m* h (166)	245.00	3.355		25.129	−43.340
MoAl_12_	WAl_12_	*cI*26	*Im*-3 (204)	436.23	7.584			−57.303
MoAl_5_	MoAl_5_	*hR*36	*R*-3*c* h (167)	558.49	4.952		26.296	−31.001
Mo_4_Al_17_	Mo_4_Al_17_	*mS*84	*C*121 (5)	1305.85	9.187	4.939	28.974	−112.563
Mo_3_Al_8_	Mo_3_Al_8_	*mS*22	*C*12/*m*1 (12)	334.46	9.235	3.653	10.091	−66.170
Mo_3_Al	Cr_3_Si	*cP*8	*Pm*-3*n* (223)	123.48	4.980			−37.228
Sc_2_Al	Ni_2_In	*hP*6	*P*6_3_/*mmc* (194)	128.50	4.902		6.176	−17.458
ScAl	CsCl	*cP*2	*Pm*-3*m* (221)	38.75	3.384			−10.973
ScAl	CrB	*oC*8	*Cmcm* (63)	81.00	3.338	11.101	4.371	−10.892
ScAl_2_	MgCu_2_	*cF*24	*Fd*-3*m* (227)	109.50	3.797			−15.277
ScAl_3_	AuCu_3_	*cP*4	*Pm*-3*m* (221)	69.25	4.107			−19.383
MoC	TiP	*hP*8	*P*6_3_/*mmc* (194)	84.84	3.016		10.768	−19.821
MoC	NaCl	*cF*8	*Fm*-3*m* (225)	21.06	4.383			−19.640
MoC	η-MoC	*hp*12	*P*6_3_/*mmc* (194)	126.16	3.074		15.401	−19.747
MoC	WC	*hp*2	*P*-6*m*2 (187)	21.00	2.928		2.829	−20.241
Mo_3_C_2_	Cr_3_C_2_	*oP*20	*Pnma* (62)	228.19	6.064	2.974	12.654	−50.938
Mo_2_C	β׳׳-Mo_2_C	*hP*3	*P*-3*m*1 (164)	38.06	3.068		4.669	−31.064
Mo_3_C	Fe_3_C	*oP*16	*Pnma* (62)	215.87	5.540	7.559	5.159	−40.423
Sc_2_C	Ti_2_C	*cF*48	*Fd*-3*m* (227)	852.33	9.481			−23.266
Sc_4_C_3_	P_4_Th_3_	*cI*28	*I*-43*d* (220)	188.75	7.227			−56.419
ScC_0.875_	NaCl	*cF*8	*Fm*-3*m* (225)	208.70	4.708			−14.923
ScC	NaCl	*cF*8	*Fm*-3*m* (225)	25.70	4.685			−15.840
Sc_3_C_4_	Sc_3_C_4_	*tP*70	*P*4/*mnc* (128)	851.50	7.515		15.076	−58.764
Mo_3_AlC	CaTiO_3_	*cP*5	*Pm*-3*m* (221)	71.70	4.154			−45.341
Mo_3_Al_2_C	Mo_3_Al_2_C	*cP*24	*P*4_1_32 (213)	327.20	6.891			−50.299
Mo_3_Al_2_C_0.9375_	Mo_3_Al_2_C	*cP*24	*P*4_1_32 (213)	1303.30	6.881			−49.691
Mo_3_Al_2_C_0.875_	Mo_3_Al_2_C	*cP*24	*P*4_1_32 (213)	648.29	6.869			−49.078
Mo_3_Al_2_C_0.875_	Mo_3_Al_2_C	*cP*24	*P*4_1_32 (213)	1296.87	6.870			−49.069
Mo_3_Al_2_C_0.75_	Mo_3_Al_2_C	*cP*24	*P*4_1_32 (213)	321.10	6.848			−47.844
Mo_2_AlC	Cr_2_AlC	*hP*8	*P*6_3_/*mmc* (194)	107.46	3.031		13.505	−35.292
Mo_3_AlC_2_	Ti_3_SiC_2_	*hP*12	*P*6_3_/*mmc* (194)	151.49	3.072		18.541	−54.830
Mo_4_AlC_3_	Ti_4_AlN_3_	*hP*16	*P*6_3_/*mmc* (194)	196.50	3.117		23.358	−74.552
(Mo_2/3_Sc_1/3_)_2_AlC	(Mo_2/3_Sc_1/3_)_2_AlC	mS48	*C*2/*c* (15)	689.78	9.367	5.427	13.961	−33.308
ScAl_3_C_3_	ScAl_3_C_3_	*hP*14	*P*6_3_/*mmc* (194)	164.34	3.362		16.789	−47.703
Sc_3_AlC	CaTiO_3_	*cP*5	*Pm*-3*m* (221)	84.90	4.395			−35.023
Sc_2_AlC	Cr_2_AlC	*hP*8	*P*6_3_/*mmc* (194)	141.75	3.296		15.065	−27.385
Sc_3_AlC_2_	Ti_3_SiC_2_	*hP*12	*P*6_3_/*mmc* (194)	199.00	3.317		20.885	−43.406
Sc_4_AlC_3_	Ti_4_AlN_3_	*hP*16	*P*6_3_/*mmc* (194)	248.50	3.296		26.414	−59.294

**Table 3:** Rietveld refinement of Mo_2_ScAlC_2_. The identified phases and their respective weight percentages according to the Rietveld refinement of the XRD pattern are: 1. Mo_2_ScAlC_2_ (73.9(0) wt.%), Mo_2_C (14.1(8) wt.%), A1_2_O_3_ (7.4(0) wt.%), Mo_3_Al_2_C (3.5(0) wt.%) and, Mo_3_Al (1.0(2) wt.%), the total χ^2^ is 10.50.

Space group	*P6*_*3*_*/mmc* (#194)
*a* (Å)	3.0334(8)
*b* (Å)	3.0334(8)
*c* (Å)	18.7750(0)
*α*	90.000
*β*	90.000
*γ*	120.000
Mo	4f (0.3333(3) 0.6666(7) 0.1363(2))
	Occupancy of Mo=4.00(0) and Sc=0.00(0)
Sc	2a (0.0000 0.0000 0.0000)
	Occupancy of Sc=1.83(4) and Mo=0.16(6)
Al	2b (0.0000 0.0000 0.2500) Occupancy of Al=2.00
C	4f (0.6666(7) 0.3333(3) 0.06825(5)) Occupancy of C=4.00

## References

[1] Meshkian R., Tao Q., Dahlqvist M., Lu J., Hultman L., Rosen J. (2016). Theoretical stability and materials synthesis of a chemically ordered MAX phase, Mo_2_ScAlC_2_, and its two-dimensional derivate Mo_2_ScC_2_ MXene. Acta Mater..

[2] Anasori B., Dahlqvist M., Halim J., Moon E.J., Lu J., Hosler B.C., Caspi E.N., May S.J., Hultman L., Eklund P., Rosén J., Barsoum M.W. (2015). Experimental and theoretical characterization of ordered MAX phases Mo_2_TiAlC_2_ and Mo_2_Ti_2_AlC_3_. J. Appl. Phys..

[3] Blöchl P.E. (1994). Projector augmented-wave method. Phys. Rev. B.

[4] Kresse G., Joubert D. (1999). From ultrasoft pseudopotentials to the projector augmented-wave method. Phys. Rev. B.

[5] Kresse G., Hafner J. (1993). Ab initio molecular dynamics for liquid metals. Phys. Rev. B.

[6] Kresse G., Furthmüller J. (1996). Efficiency of ab-initio total energy calculations for metals and semiconductors using a plane-wave basis set. Comput. Mater. Sci..

[7] Kresse G., Furthmüller J. (1996). Efficient iterative schemes for ab initio total-energy calculations using a plane-wave basis set. Phys. Rev. B.

[8] Perdew J.P., Burke K., Ernzerhof M. (1996). Generalized gradient approximation made simple. Phys. Rev. Lett..

[9] Monkhorst H.J., Pack J.D. (1976). Special points for Brillouin-zone integrations. Phys. Rev. B.

[10] Zunger A., Wei S.H., Ferreira L.G., Bernard J.E. (1990). Special quasirandom structures. Phys. Rev. Lett..

[11] Dahlqvist M., Alling B., Abrikosov I.A., Rosén J. (2010). Phase stability of Ti_2_AlC upon oxygen incorporation: a first-principles investigation. Phys. Rev. B.

[12] Dahlqvist M., Alling B., Rosén J. (2010). Stability trends of MAX phases from first principles. Phys. Rev. B.

[13] Eklund P., Dahlqvist M., Tengstrand O., Hultman L., Lu J., Nedfors N., Jansson U., Rosén J. (2012). Discovery of the ternary nanolaminated compound Nb_2_GeC by a systematic theoretical-experimental approach. Phys. Rev. Lett..

[14] Ingason A.S., Mockute A., Dahlqvist M., Magnus F., Olafsson S., Arnalds U.B., Alling B., Abrikosov I.A., Hjörvarsson B., Persson P.O.Å., Rosen J. (2013). Magnetic self-organized atomic laminate from first principles and thin film synthesis. Phys. Rev. Lett..

[15] Togo A., Oba F., Tanaka I. (2008). First-principles calculations of the ferroelastic transition between rutile-type and CaCl_2_-type SiO_2_ at high pressures. Phys. Rev. B.

[16] Togo A., Tanaka I. (2015). First principles phonon calculations in materials science. Scr. Mater..

[17] Henkelman G., Arnaldsson A., Jónsson H. (2006). A fast and robust algorithm for Bader decomposition of charge density. Comput. Mater. Sci..

[18] Rietveld H.M. (1969). A profile refinement method for nuclear and magnetic structures. J. Appl. Crystallogr..

